# DO WHAT I SAY, NOT WHAT I DO? CHALLENGING CONTEMPORARY WISDOM ABOUT RESIDENCE IN OLD AGE

**DOI:** 10.1093/geroni/igz038.818

**Published:** 2019-11-08

**Authors:** Graham D Rowles

**Affiliations:** University of Kentucky, Lexington, Kentucky, United States

## Abstract

An environmental gerontologist teaching about “aging and environment” for 30 years, I had it all figured out. Downsize and divest, stair free environment, universal design, environmental centralization, location close to services. At 60, I was consulting with an architect to design an age friendly home and searching for a level plot. But then, serendipitously, we found our current home, a residence defying all of these principles. An irresponsible denial of aging and pending frailty? Thirteen years later, I’m not so sure. In this presentation, I explain why, focusing on processes of “making and remaking home” and “being in place” that have been central themes in my research career. I explore ways in which as my wife and I grow older, harnessing modern technologies, newly emergent living arrangements and contemporary communication, we anticipate being able to age in place far longer than has been possible for previous generations.

